# Multifidus dysfunction and restorative neurostimulation: a scoping review

**DOI:** 10.1093/pm/pnad098

**Published:** 2023-07-13

**Authors:** Vinicius Tieppo Francio, Benjamin D Westerhaus, Alexios G Carayannopoulos, Dawood Sayed

**Affiliations:** Department of Physical Medicine & Rehabilitation, The University of Kansas Medical Center, Kansas City, KS 66160, United States; Department of Anesthesiology and Pain Medicine, The University of Kansas Medical Center, Kansas City, KS 66160, United States; Cantor Spine Institute at the Paley Orthopedic & Spine Institute, West Palm Beach, FL 33407, United States; Department of Neurosurgery and Neurology, Warren Alpert Medical School of Brown University, Providence, RI 02903, United States; Department of Anesthesiology and Pain Medicine, The University of Kansas Medical Center, Kansas City, KS 66160, United States

**Keywords:** multifidus, restorative neurostimulation, lumbar medial branch nerve, neuromodulation, peripheral nerve stimulation, chronic low back pain, sensorimotor control, neuromuscular control

## Abstract

**Objective:**

Chronic low back pain (CLBP) is multifactorial in nature, with recent research highlighting the role of multifidus dysfunction in a subset of nonspecific CLBP. This review aimed to provide a foundational reference that elucidates the pathophysiological cascade of multifidus dysfunction, how it contrasts with other CLBP etiologies and the role of restorative neurostimulation.

**Methods:**

A scoping review of the literature.

**Results:**

In total, 194 articles were included, and findings were presented to highlight emerging principles related to multifidus dysfunction and restorative neurostimulation. Multifidus dysfunction is diagnosed by a history of mechanical, axial, nociceptive CLBP and exam demonstrating functional lumbar instability, which differs from other structural etiologies. Diagnostic images may be used to grade multifidus atrophy and assess other structural pathologies. While various treatments exist for CLBP, restorative neurostimulation distinguishes itself from traditional neurostimulation in a way that treats a different etiology, targets a different anatomical site, and has a distinctive mechanism of action.

**Conclusions:**

Multifidus dysfunction has been proposed to result from loss of neuromuscular control, which may manifest clinically as muscle inhibition resulting in altered movement patterns. Over time, this cycle may result in potential atrophy, degeneration and CLBP. Restorative neurostimulation, a novel implantable neurostimulator system, stimulates the efferent lumbar medial branch nerve to elicit repetitive multifidus contractions. This intervention aims to interrupt the cycle of dysfunction and normalize multifidus activity incrementally, potentially restoring neuromuscular control. Restorative neurostimulation has been shown to reduce pain and disability in CLBP, improve quality of life and reduce health care expenditures.

## Introduction

Low back pain (LBP) is the leading cause of years lived with disability globally with a prevalence of an estimated 568 million people worldwide. LBP is the most common musculoskeletal condition with a lifetime prevalence as high as 65%–80%, affecting 52 million people in the United States. There is a huge cost to treating LBP in the United States with American healthcare spending totaling $135 billion annually.^1–3^ LBP can be temporally categorized as acute, subacute, or chronic, and further categorized as axial, radicular, and/or referred pain.^4–6^ While most acute LBP events are self-limited, an average of 35% can lead to subacute and/or chronic pain.^7^ An individual with LBP may experience episodic flare-ups over time, where increasing frequency increases the likelihood of chronic pain by 15%–20%.^3^^,^^6–9^ Estimates suggest that 15%–20% of LBP events have an identified cause, leaving up to 80–85% without a clear etiology commonly labeled as non-specific LBP.^4–6^^,^^10–12^ Management of LBP can be challenging, and unfortunately, treatment outcomes are variable. Chronic low back pain (CLBP) has become the most expensive medical problem in the United States.^1–4^^,^^11–12^ As such, we must understand distinctive features of pain neurobiology, such as neuropathic, nociceptive, and nociplastic pain to try to customize individual treatment and optimize outcomes.

The International Association for the Study of Pain (IASP) defines neuropathic pain as central or peripheral pain “caused by a lesion or disease of the somatosensory nervous system” and occurs as a result of abnormal neural activity. It is commonly characterized as burning, electric, and/or shooting pain, which follows a neuroanatomically plausible distribution with or without motor or sensory deficits. Nociceptive pain, as defined by the IASP, is “pain that arises from actual or threatened damage to non-neural tissue and is due to the activation of nociceptors.”^13–15^ Typically, nociceptive pain has a clear and proportional relationship to movement-based factors and predictably occurs with specific activities or postures.^4–5^^,^^13–16^ The IASP has defined nociplastic pain as “pain that arises from altered nociception despite no clear evidence of actual or threatened tissue damage causing the activation of peripheral nociceptors or evidence for disease or lesion of the somatosensory system causing the pain.” In turn, this may elicit central sensitization or widespread hypersensitivity, which may not be directly associated to tissue damage. Usually, there is a non-linear and aberrant relationship with movement, often with disproportionate, non-mechanical, diffuse, unpredictable patterns of pain provocation or fear-avoidance/kinesiophobic behavior.^13–17^

CLBP is a nebulous symptom and often the underlying etiology is nonspecific, complex, and multifactorial with combined pain generators that may lead to challenging treatments and limited success. As such, for many decades there has been a focus in identifying potential etiologies that may play a role in the recurrence and maintenance of CLBP with respective treatments that could optimize treatment outcomes.^8–10^ Functional spinal instability resulting from multifidus muscle dysfunction secondary to arthrogenic muscle inhibition (AMI) and loss of neuromuscular control has emerged as an important functional etiology. This may be one important driving factor in the maintenance and recurrence of LBP chronicity.^18–25^ It is important to note that a “functional etiology” refers to the function of movement, posture, and neuromuscular control, rather than a structural pathology, such as in structural spinal instability secondary to degenerative spondylosis, spondylolisthesis or fracture.^26–28^ With the recognition of multifidus dysfunction as a proposed source of non-specific CLBP, restorative neurostimulation has emerged as a disease-modifying novel implantable neurostimulator that may arrest this cycle by overriding multifidus inhibition with efferent lumbar medial branch nerve (LMBN) stimulation resulting in repetitive multifidus contractions, which may restore neuromuscular control, spinal stability and ultimately lead to improvements in pain and function.^11^^,^^19–21^^,^^29–36^ Yet there is a gap in the literature comprehensively discussing the scientific background on this potential link between loss of neuromuscular control, AMI, multifidus dysfunction, functional instability, and CLBP. Furthermore, there is a current need in the literature discussing how restorative neurostimulation differs from other neuromodulation therapies, their anatomical targets and mechanisms of action (MOA).

Therefore, in this scoping review we aim to define the functional pathophysiological cascade of how multifidus dysfunction may play a role in LBP recurrence and chronicity, contrasting to other CLBP etiologies. Then we discuss treatment options by exploring the distinctions between traditional and restorative neurostimulation therapy.

## Methods

**Figure 1.**
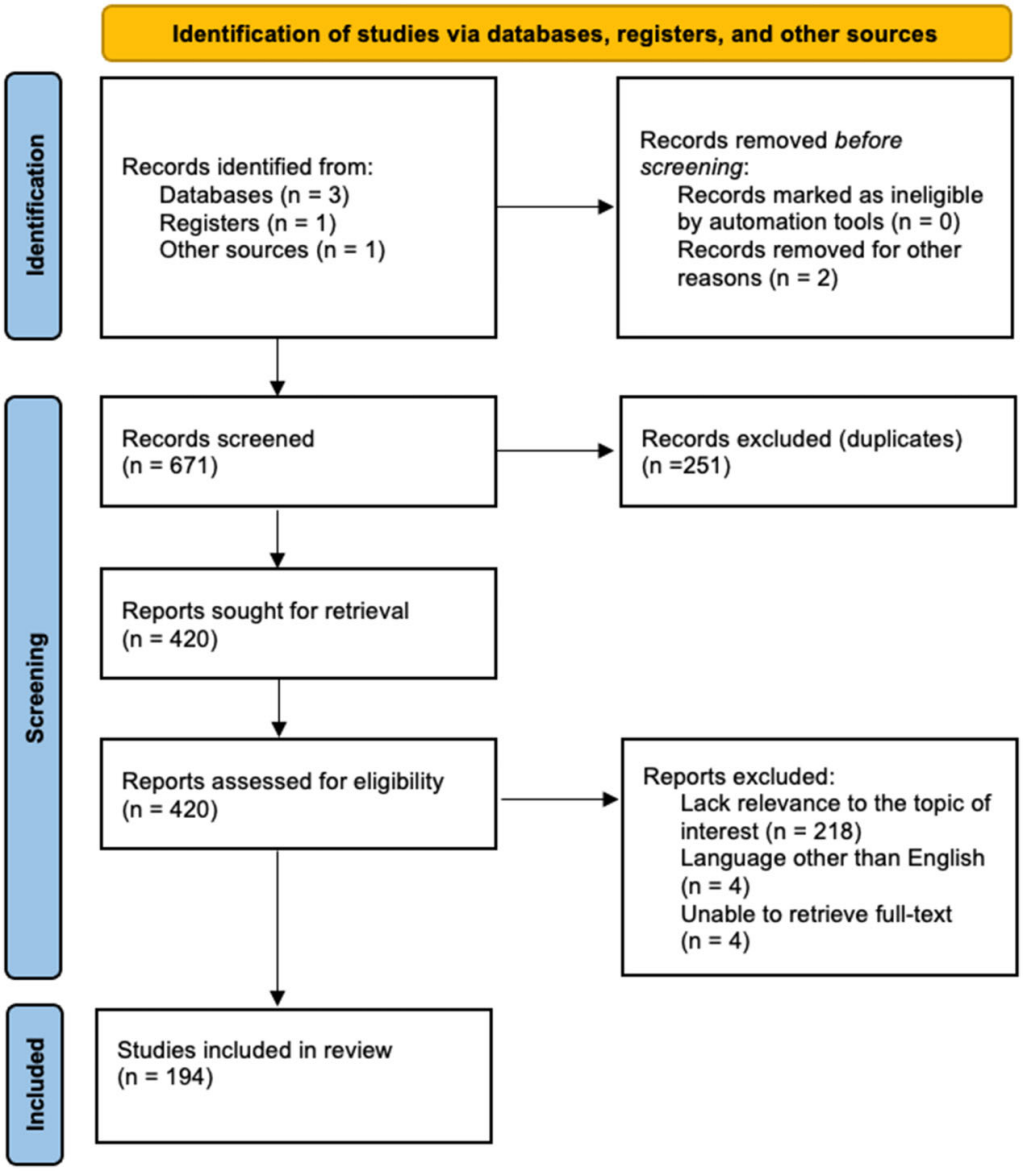
PRISMA-ScR methodology and results flowchart with identification, screening, eligibility and inclusion and exclusion process. Adapted from: Tricco et al 2018.^37^

This scoping reviewed followed the Preferred Reporting Items for Systematic Reviews and Meta-Analysis for scoping reviews (PRISMA-ScR) (Figure 1) and the step-by-step process for scoping review based on Peters et al. JBI manual for evidence synthesis.^37–38^ The scoping review protocol was registered on the Open Science Framework. A scoping review is a type of literature review that aims to map the evidence available with key concepts on a particular topic. This is particularly useful when the literature is heterogeneous and diverse, and there is a need to identify gaps in the literature and answer these with foundational knowledge to guide future studies.^37–38^ Our first step was to formulate clear objectives and specific research questions to guide the scoping review process to address the gaps in the literature, followed by a framework design to identify the population, context, and concept approach of eligibility criteria. Then, we conducted a comprehensive search strategy to identify relevant studies in electronic databases, and later proceeded to screen the identifiable studies based on predetermined inclusion and exclusion criteria in a nonbiased fashion. Data were extracted from the eligible studies, synthesized, and presented in a clear and concise manner using a descriptive format into specific themes within this review.

### Search strategy and terms

A comprehensive search of electronic databases (MEDLINE, PubMed, Embase, and Cochrane Library) was conducted for studies published in English language since inception to February 2023 with the first search performed in June 2022 and a repeat search in April 2023. We searched for meta-analysis, systematic reviews, narrative, and clinical reviews, randomized and non-randomized trials, prospective and retrospective studies, and case series. Case reports, letters, editorials, conference abstracts and commentaries were excluded. The following combination of subject headings and keyword search terms were utilized: “multifidus,” “restorative neurostimulation.” “lumbar medial branch nerve,” “neuromodulation,” peripheral nerve stimulation,” “sensorimotor control,” “neuromuscular control,” and “chronic low back pain.” The search strategy was developed by the authors in consultation with a research librarian.

### Eligibility criteria

A population, concept, and context approach was followed. No patient data were involved in this scoping review. The population of interest was subjects with CLBP associated with multifidus dysfunction. The core concept of importance was to define how neuromuscular control loss and AMI may lead to multifidus dysfunction and functional lumbar instability, which in turn is theoretically presumed to contribute to CLBP, while contrasting with other LBP etiologies. Furthermore, we summarized how restorative neurostimulation may address this etiology, briefly comparing with traditional neuromodulation therapies. The context of interest was to provide a state-of-the-art comprehensive review of the literature to serve as a foundational reference on multifidus dysfunction and restorative neurostimulation to help enhance comprehension of this complex functional pathophysiological process and optimize practitioners' understanding on why and when to utilize this intervention. Thereby, improved awareness of this theme may lead to optimal selection and therapy application, thus avoiding overutilization, reimbursement reduction, and sustaining long-term durable outcomes with exemplary safety.

### Article selection and inclusion

We conducted a comprehensive search of electronic databases stated above. After the initial search, duplicates were removed, and titles and abstracts of articles were screened. All eligible studies identified in the search were independently appraised by two reviewers in a standardized, unblinded fashion, using the same strategy to ensure proper cross-checking of the results with the PRISMA-ScR method/checklist to reduce selection bias and standardize inclusion and exclusion criteria. Any disagreement between the two screeners was flagged for resolution, mediated by a third and fourth author independently. Eligible full-text articles of potential interest were fully reviewed following the population, concept, and context eligibility criteria. Studies that investigated the relationship between the multifidus muscle and CLBP, studies that assessed multifidus function and structure in subjects with CLBP, studies that evaluated multifidus assessment in CLBP, studies that examined interventions aimed at improving multifidus function in individuals with and without CLBP were included. Other studies were included to supplement the core concept and answer the research questions.

### Data extraction

Data extraction focused on capturing key information to answer the research questions and the study objectives. The data from the selected studies were comprehensively reviewed and organized into subsections within the results section of this scoping review. As designated by scoping review methodology, the data extracted were utilized to map the literature on this subject area, identifying key principles to answer current gaps in the literature. A quality assessment of the included studies was not performed, as scoping reviews aim to provide a broad overview of the literature, rather than a critical appraisal of individual studies.^37–38^

## Results

We found 671 citations through our initial online database search. After removal of duplicates, 420 records remained and were screened as per our selection criteria. Of these, 218 were excluded because of lack of relevance to the topic of interest to answer the proposed research questions and study objectives. An additional 8 studies were excluded due to language other than English and unable to retrieve full text. The remaining 194 full-text articles were included, comprising of meta-analysis, systematic reviews, clinical and narrative reviews, randomized clinical trials, prospective, retrospective, observational clinical studies, population based and basic science studies. Key findings were presented along the results section in a thematic organization with figures and in a descriptive format contrasting the literature to highlight critical and emerging principles related to multifidus dysfunction and restorative neurostimulation.

### Definition of concepts

#### The multifidus muscle

The role of the lumbar multifidus muscle (MM) is well established as the strongest spine stabilizer, accounting for more than two-thirds of spinal stiffness, providing intersegmental spinal stability, withstanding compressive loading and preventing shear forces of the lumbar spine.^25^^,^^39–41^ The deep multifidus is a group of medially oriented, short fibers that provide compression to maintain intersegmental spinal control by attaching superiorly to the laminae of L1–L5 and inferiorly to the mamillary process of vertebrae one level below.^39^^,^^42^ The intermediate multifidus fascicle is longer, spanning across three to four spinal segments and carries mixed stabilizing and mobilizing functions. Finally, the superficial multifidus is progressively longer with fibers crossing up to five segments and is responsible for generating end-range spinal extension in concert with the erector spinae group (longissimus and iliocostalis).^42–44^ The middle branches of the dorsal ramus of the LMBN are found deep into the lumbar intertransversarii muscles and are responsible to innervate the MM, however there is debate if the muscle is segmentally or polysegmentally innervated.^45–48^ Importantly, a unique feature of MM architecture is that it possesses a greater cross-sectional area than the other spinal muscles. As such, the bilateral multifidi exert large compressive force over a small excursion, yielding stabilization of spinal segments rather than gross spinal movement. Additionally, deep MM architecture contains a dense number of muscle spindles, which are strategically positioned combined with joint mechanoreceptors to provide proprioceptive feedback to the central nervous system, playing a critical role in neuromuscular control and spinal stability.^25^^,^^39–44^

#### Spinal stability

**Figure 2.**
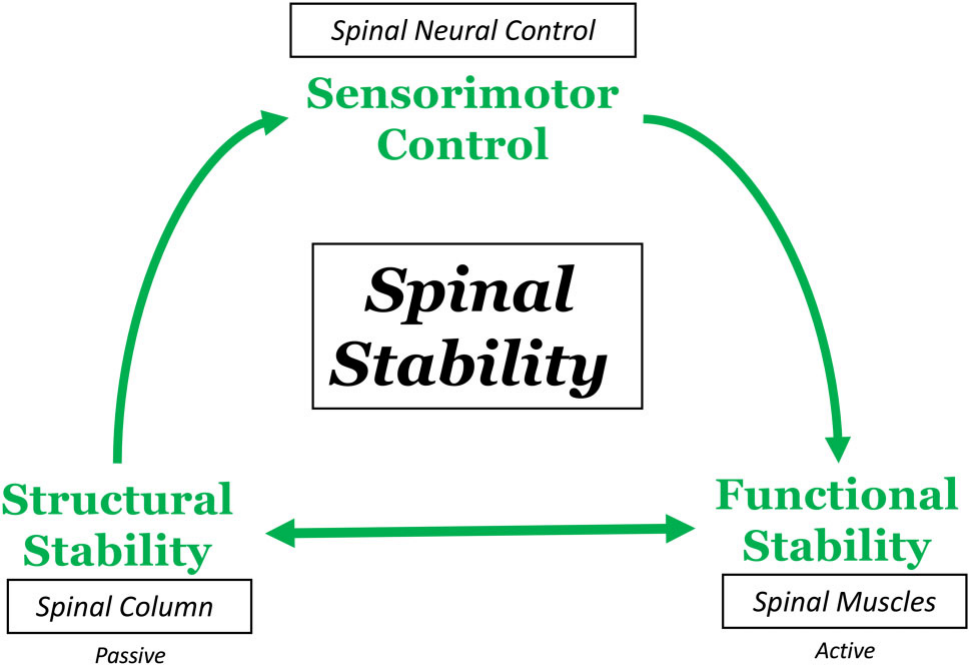
Updated diagram based on Panjabi’s landmark^49–51^ and expanded models^26–28^^,^^55–56^ and, denoting the complex interplay among the spinal column (passive subcomponent), the spinal muscles (active subcomponent) and sensorimotor control (spinal neural control) to maintain spinal stability.

Spinal instability is a clinical term that may encompass structural and functional components.^26^ Structural instability is based on radiographic findings suggesting an increase in end range of motion (hypermobility) with structural abnormalities that may be amenable to surgical stabilization.^26–27^ Conversely, functional instability is more likely related to a loss in neuromuscular control leading to a decrease in spinal stiffness and mid-range intersegmental aberrant motion, which is clinically diagnosed with physical exam maneuvers that assess the neuromuscular kinetic chain for aberrant movement patterns.^27–28^ Importantly, structural and functional instability may present in combination, as first described by Panjabi’s landmark model of spinal stability, that identified the spinal column, spinal muscles, and spinal neural control unit as a stabilizing system.^49–51^ Years later, further research expanded on this model and demonstrated how spinal joint mechanoreceptors and adjacent muscle spindles afferents contribute to spinal stability by providing kinesthetic perception to the sensorimotor cortex.^52–54^ This framework was collective identified as sensorimotor control (Figure 2).^55–56^

#### Sensorimotor and neuromuscular control

The sensorimotor system incorporates all the afferent, efferent, central integration and processing components involved in maintaining functional joint stability during movement.^57^ Dynamic contributions arise from feedforward and feedback neuromuscular control over skeletal muscles adjacent to joints. As such, peripheral mechanoreceptors are the most important components from a musculoskeletal standpoint, yet the interpretation of sensorimotor control is at the somatosensory cortex.^55–57^ Neuromuscular control is a term related to sensorimotor control that refers to the neuromusculoskeletal kinetic chain collectively functioning in harmony to enable dynamic stability and movement patterns under control of the central nervous system.^55^^,^^57^ Specifically, from a joint stability perspective, neuromuscular control has been defined as the unconscious activation of dynamic restraints occurring in preparation for and in response to joint motion and joint loading for the purpose of maintaining and restoring functional joint stability.^57^

#### Altered neuromuscular control

Altered neuromuscular control has been proposed one potential contributing factor in the pathophysiological process of CLBP. This theory suggests that disruptions in the somatosensory feedback between muscles and joints afferents can lead to loss of neuromuscular control.^18–19^^,^^22–24^^,^^57–59^ However, it is important to note that altered neuromuscular control is just one of many proposed contributors of non-specific CLBP and often other structural pathologies may be present.^4^^,^^9^^,^^18–19^^,^^22^^,^^58–59^

#### Arthrogenic muscle inhibition

Arthrogenic muscle inhibition (AMI) is defined as altered muscle activity due to neural inhibition secondary to a change in articular sensory discharge.^60–61^ AMI can be a protective mechanism via spinal reflex pathway proposed to take effect in altered sensorimotor control from spinal joint mechanoreceptors acting on the motor pool Ib inhibitory interneurons resulting in adjacent muscle inhibition.^62–64^ AMI is a well-recognized phenomenon clinically confirmed under ultrasonography and electromyography (EMG). AMI has been shown to result in decreased muscle activity, fatigue, and spasm in adjacent muscles.^62–63^^,^^65–68^

#### Multifidus dysfunction

Multifidus dysfunction is a clinical diagnosis that manifests as impaired multifidus activity/muscle inhibition, resulting in loss of spinal stiffness in the neutral zone, which may enhance an environment of relatively functional instability.^19^^,^^24–25^ MM dysfunction has been proposed to result from spinally induced arthrogenic inhibition in the setting of altered neuromuscular control.^19^^,^^24^^,^^66^ When the MM is inhibited, it yields reduced voluntary recruitment and limited motor units on EMG with replacement of tonic activation with phasic bursts of activity and muscle fibers transformation (slow type I to fast type II), which may ultimately result in muscle atrophy, aberrant co-contraction, and fatigue-spasm cycles.^69–76^ MM dysfunction may continue even after the symptom of back pain have resolved, and the persistent loss of neuromuscular control may contribute to the high recurrence rate of LBP.^19^^,^^25^^,^^41^^,^^77–80^

#### Multifidus dysfunction and loss of neuromuscular control

Over time, these changes observed in the structure and function of the MM are believed to result in altered movement behaviors leading to cortical reorganization and neuromuscular control loss.^19^^,^^58–59^^,^^81–84^ At first, changing movement patterns may be protective to prevent worsening pain and reaggravation of injury. These may be beneficial in the short-term but might carry negative consequences over time, potentially resulting in fear-avoidance behavior that may contribute to LBP chronicity.^81^^,^^85–87^ If neuromuscular control loss endures, subjects may alter their movement patterns unconsciously secondary to kinesiophobia, which may lead to reduced mobility, worsening pain, and altered central processing, thereby amplifying pain perception.^79^^,^^87–90^ These reflect neuroplastic changes in neuromuscular control loss, which have been associated to chronic pain states supporting the hypothesis that it might play a key role in LBP chronicity.^81–88^^,^^91–94^

### Multifidus dysfunction and chronic low back pain

**Figure 3.**
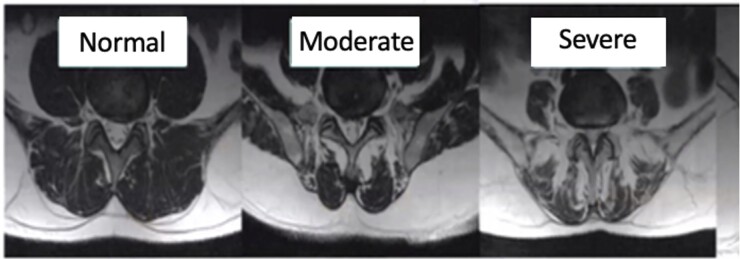
Magnetic resonance images demonstrating progressive multifidus fat infiltration from normal to moderate to severe.

Numerous studies have demonstrated an association between the duration of LBP symptoms and multifidus dysfunction with or without structural changes such as fat infiltration, atrophy, and diminished cross-sectional area (Figure 3). However, it is critical to note that multifidus fat infiltration and pain can occur independently of atrophy, and the evidence remains mixed.^91–109^ Studies have reported that a high multifidus fat index has been associated with facet joint and intervertebral disc degeneration, spondylolisthesis, and degenerative stenosis. Furthermore, multifidus atrophy has been associated to suboptimal spinal alignment, such as increased lumbar lordosis, thoracic kyphosis, and sacral angle.^110–126^ Furthermore, basic science, animal and human studies have proposed an interaction between pro-inflammatory cytokines with changes in multifidus architecture. These have reported that a dysregulation of interleukin 1, interleukin 6, transforming growth factor, macrophages, and tumor necrosis factor contribute to differentiation of fibroblasts and pre-adipocytes in multifidus atrophy.^127–133^

Overall, the literature seems to favor a positive correlation between multifidus atrophy, muscle dysfunction, and LBP recurrency and chronicity. However, it is important to note that this is variable among individuals and during distinctive functional tasks, as noted by advanced imaging and/or EMG findings.^41^^,^^72^^,^^77^^,^^97–100^^,^^131–138^ There are studies that have not found a significant association of multifidus atrophy and LBP, suggesting that the evidence is mixed due to heterogenicity, differences in methodology, cofounding variables, and distinctive populations.^101^^,^^106–108^^,^^125^^,^^139–145^

### Treatment options for multifidus dysfunction and loss of neuromuscular control

Similar to other etiologies, CLBP associated with multifidus dysfunction should follow an evidence-based treatment algorithm focused on conservative methods first. These include activity modifications, concurrent medication management to provide temporary symptom relief to facilitate participation in an active physical therapy program.^146^ When conservative therapies are insufficient to offer durable symptom relief, restorative neurostimulation of the LMBN may be considered.

#### Motor control exercises and patient education

General spinal stabilization exercises have demonstrated inconsistent effects on the lumbar multifidus muscle structure and function in patients with CLBP.^147–149^ Motor control exercises may help reduce LBP disability and severity at short-term, particularly when combined with patient education.^150–155^ However, the general consensus is that there is limited evidence to support that motor control exercises is superior to other therapeutic exercises, which may be due to the difficulty in voluntary isolation of the multifidus, particularly in longstanding pain states with altered movement behaviors and cortical remodeling.^81–88^^,^^156^

#### Restorative neurostimulation of the lumbar medial branch nerve

Restorative neurostimulation is a novel permanently implanted peripheral nerve stimulation (PNS) system that targets the motor fibers of the dorsal rami of the L2 LMBN, which through efferent neurostimulation results in bilateral multifidus muscle contractions.^11^^,^^21^^,^^29^ Currently, this is the only Food and Drug Administration (FDA) approved device to treat CLBP in the setting of multifidus dysfunction (ReActiv8 system; Mainstay Medical, San Diego, CA, USA).^29–30^ Several high-quality clinical studies have been published reporting long-term durable improvements (up to three years) in pain, disability, better quality of life, with individual studies also reporting decreased opioid use and health care utilization reduction.^21^^,^^30–36^

## Discussion

**Figure 4.**
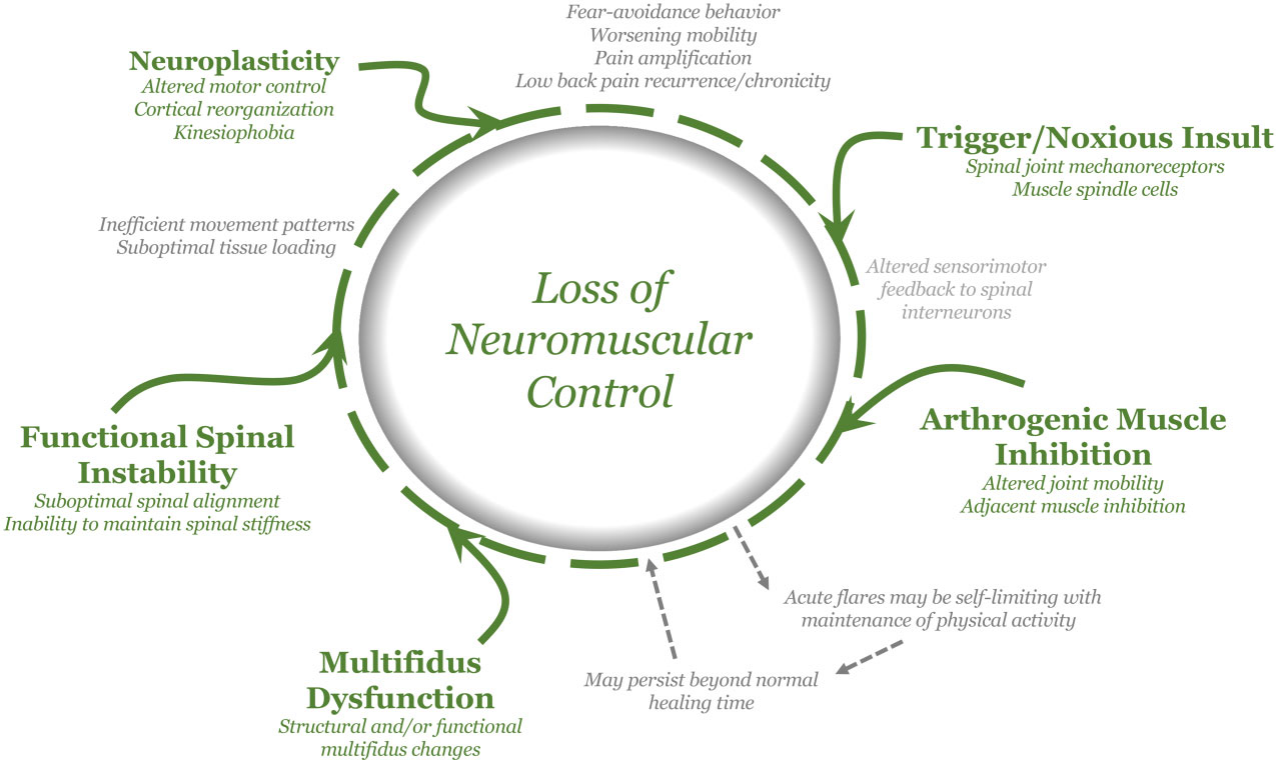
Diagram illustrating the complex interplay between altered sensorimotor control, arthrogenic muscle inhibition, multifidus dysfunction, functional spinal instability, and neuroplastic changes that may result in loss of neuromuscular control contributing to low back pain recurrency and chronicity.

This is the first study to comprehensively review the scientific literature and to assemble the current state of knowledge to delineate how the principles of altered neuromuscular control and AMI may lead to multifidus dysfunction, functional lumbar instability and CLBP. As such, this study aimed to provide a foundational reference to illustrate the complex functional disease-process proposedly addressed with restorative neurostimulation (Figure 4).

CLBP is often multifactorial and categorized as nociceptive, neuropathic or nociplastic.^4^^,^^13^ However, such narrowed categorization to one type does not represent the true prevalence in clinical practice. In fact, the majority of CLBP is a mixed pain syndrome with an overlap of pain types and a constellation of symptoms that might occur in a continuum cycle. Ultimately, such clinical picture may be labeled by clinicians and researches as nonspecific CLBP, however this has not yet been recognized by the IASP.^13–14^^,^^157^ For decades there has been a focus in exploring, understanding and identifying this clinical picture. Basic sciences, clinical and population-based studies seem to support the hypothesis that multifidus dysfunction in the setting of loss of neuromuscular control would be one plausible etiology, however this is obviously not the only possible factor.^19^^,^^22–24^^,^^41^^,^^58–59^^,^^77^^,^^79^^,^^85^^,^^92^^,^^98^^,^^158^

By way of illustration, consider a clinical scenario with a nonspecific event that triggers a noxious insult to spinal joint mechanoreceptors and/or paraspinal muscle spindles that can result in joint or muscle overload. In turn, this can lead to an influx of aberrant mechanoreceptive input towards the spinal cord interneurons. Automatically within the spinal pathway, a spinally-induced AMI reflex may occur to subconsciously protect the spinal joint by limiting segmental spinal motion. This limitation in movement further decreases mechanoreceptive discharge to surrounding muscles leading to reflexive tonic contraction and potentially a painful cycle of muscle spasm and muscle overload. Usually, such acute events are self-limiting, and the deleterious effects may be lessened with maintenance of physical activity, despite acute pain. However, if altered sensorimotor feedback persist beyond normal healing time, it may lead to loss of neuromuscular control. Over time if uncorrected, this may lead to persistent multifidus inhibition/dysfunction and the inability to maintain spinal stiffness in the neutral zone, resulting in an augmented environment for functional lumbar instability. Consequently, this may express symptomatologically as nonspecific LBP.

Altered spinal proprioception resulting in poor posture and loss of neuromuscular control has been documented in subjects with CLBP.^53^^,^^91–93^^,^^158–166^ Multifidus dysfunction, diminished cross-sectional area, and muscle fibers transformation results in inefficient activation, which has been linked to suboptimal spinal alignment and functional instability.^75–79^^,^^87^^,^^98^^,^^102^^,^^111^^,^^115–116^^,^^120^ Over time, these changes observed in the structure and function of the multifidus muscle are believed to result in worsening of movement patterns and cortex reorganization, which may translate as disproportionate pain.^81–86^

Multifidus dysfunction may present primarily with a nociceptive-mechanical pain pattern, with pain usually movement or position related, and aggravated by trivial activities and small movement tasks, such as returning to an erect posture from bending, leaning over a sink, unloading the dishwasher, reaching out for an item, lower extremity dressing, and so forth. Pain patterns may be persistent with sustained postures, such as prolonged sitting, standing, walking, driving, and pain may abide with use of a back brace or back rest on a chair due to limited ability to maintain spinal stability.^29–30^ Usually, LBP is predominantly axial, however non-neuropathic leg pain with referral into the sacroiliac or gluteal region and proximal to the knee is plausible.^30–31^^,^^34^ As previously mentioned, most CLBP have a mixed pain picture.^157^ Multifidus dysfunction may present similarly with a mixed pain syndrome depending on the time-to-effect of neuroplastic changes secondary to the loss of neuromuscular control, however ideally pain is primarily of mechanonociceptive without neuropathic or with limited nociplastic features. Furthermore, concomitant structural spinal pathologies such as degenerative spondylosis and stenosis, spondylolisthesis and disc herniation, may be present concurrently with multifidus muscle dysfunction, and these are not a contraindication to restorative neurostimulation, as evidence by randomized clinical trials, as long as there is no pathology amenable to spinal surgery.^30–31^^,^^34^

As a proposed functional etiology, it is imperative to assess CLBP beyond structural changes on diagnostic images and shift thinking to a functional perspective. Multifidus dysfunction must be diagnosed with a combination of clinical history, physical exam and/or MRI findings A physical exam to evaluate for signs of functional lumbar instability with a positive prone instability test (κ = 0.87) preferably, multifidus lift test (κ = 0.75 to 0.81) and a positive aberrant movement patterns test (κ = 0.60) have demonstrated sustained interrater reliability.^29^^,^^167–169^ Diagnostic ultrasound and EMG can help assess multifidus activity during functional movements, while MRI can help grade multifidus atrophy.^69–70^^,^^75–76^^,^^109^^,^^131–134^^,^^170^ However, the finding of muscle atrophy on MRI alone does not establish multifidus dysfunction diagnosis (since this is a functional etiology) and by itself should never be relied upon as a sole criterion for the indication for restorative neurostimulation.^30^ Importantly, MRI findings can help correlate physical examination and clinical history to corroborate diagnosis and assure that there are no concomitant structural pathology amenable to traditional spinal surgery.^30–31^^,^^34^ Commonly, these patients are not surgical candidates and a have limited response to conservative management without longitudinal improvement in pain and function. In such settings, restorative neurostimulation therapy could be considered.^29–31^^,^^171–172^

Restorative neurostimulation treatment is clinically indicated for patients with CLBP greater than 6 months of duration with diagnostic evidence of multifidus dysfunction seen on physical exam and/or on diagnostic imaging.^11^^,^^19–21^^,^^29–31^^,^^171–172^ This is an innovative neurostimulation system that has emerged as a disease-modifying therapy to restore neuromuscular control through a rehabilitative MOA rooted in the previous discussion of the pathophysiology of multifidus dysfunction and loss of neuromuscular control. This therapy is believed to restore this by an efferent neurostimulation of the dorsal rami of the LMNB, which leads to repetitive multifidus contraction that with a gradual and longitudinal accrual time to effect may override underlying AMI and restore mechanonociceptive feedback, which may normalize neuromuscular control and functional spinal stability.^11^^,^^19–21^^,^^29–34^^,^^171^ Thereby, the therapeutic goal is restorative in nature to first normalize neuromuscular function, and subsequently reduce disability and pain. Hence, the stimulation plan is long-term (months to years) and pain/disability reduction has a longitudinal gradual effect, with numerous studies reinforcing the distinctive MOA based on durable and robust sustained improvement up to 36 months.^20–21^^,^^30–36^ Because of the MOA and clinical benefits that accumulate over time, it is thought that a short trial period is unlikely to yield optimized patient selection and predict future responders.^29^

Importantly, restorative neurostimulation distinguishes itself from traditional neuromodulation in a way that treats a different etiology, targets a different anatomical site, and has a distinctive MOA. Spinal cord stimulation (SCS) has a palliative neuromodulatory effect by blocking nociceptive afferents in the dorsal columns and is traditionally indicated for CLBP neuropathic in nature.^173–178^ Meanwhile, temporary implanted (60-day) PNS devices offer primarily palliative analgesia in an afferent fashion by modulation of central and peripheral sensitization, proposed to offer symptomatic relief of intractable CLBP with underlying structural etiologies (post-surgical, post-traumatic).^179–185^ Furthermore, lumbar radiofrequency ablation is traditionally utilized for symptomatic management of facet arthropathy, a well-identifiable structural etiology of axial LBP, and offers immediate analgesia by creating a temporary ablative destruction of the LMBN.^171^^,^^186^ However, this has been hypothesized to have deleterious effects on structures supplied by the LMBN, such as the multifidus muscle. However, the evidence to support this claim is theoretically plausible, but inconclusive.^186–190^ Of note, restorative neurostimulation may be successful in the setting of prior lumbar radiofrequency ablation, as long as the procedure was performed six-months prior, and recent studies have shown positive benefits in such population.^30–31^^,^^34^

CLBP associated with multifidus dysfunction and loss of neuromuscular control must be treated with a multidisciplinary approach, given that 1 therapy alone is less beneficial in scenarios of sustained neuroplastic changes, cortical remodeling and kinesiophobia. These factors have been linked to limited and/or negative treatment outcomes. Treatment may require longer duration to restore neuromuscular control and reverse neuroplastic changes, therefore an adjunctive program with biofeedback, cognitive behavioral therapy, fear avoidance, and pain neuroscience education may be necessary to yield better participation in therapies and to optimize outcomes.^3^^,^^82–83^^,^^87–89^^,^^150^^,^^152–153^^,^^191–194^ Restorative neurostimulation may be beneficial earlier in the treatment algorithm (as an escalation to augment physical therapy program) to restore neuromuscular control, before the presence of cortical neuroplastic changes seen in chronic pain states, which have been associated to poor outcomes. In turn, this could lead to decreased need for additional interventions, reduced opioid consumption, lower psychological burden, and perhaps reduce LBP recurrence and chronicity, thereby limiting healthcare expenditures.

## Limitations

Although we followed the journal’s guidelines on scoping reviews, including the PRISMA-ScR and Peter et al.^38^ framework, this scoping review has limitations that should be considered when interpreting the discussion of the results section. Assessment of the quality of the studies was not performed due to the heterogenicity of included studies. Data synthesis and key summary of findings was presented in a descriptive format, rather than a quantitative format since this scoping review highlights a conceptual framework and does not compare the same intervention and outcomes within the same or populations. It was outside the scope of this study to extensively review procedural techniques, safety profile, and comparative clinical results since other studies have covered these topics in detail. Selection bias in the extraction process cannot be excluded; however, this was mitigated by using a systematic transparent approach following the above-cited guidelines.

## Conclusion

Multifidus dysfunction has been proposed to result from spinally induced AMI in the setting of loss of neuromuscular control. Multifidus dysfunction is a clinical diagnosis that manifests as muscle inhibition resulting in impaired muscle activity leading to loss of spinal stiffness, which may enhance an environment for relatively functional instability. Multifidus dysfunction is diagnosed by a clinical history of primarily mechanical, positional related, predominantly axial nociceptive CLBP without neuropathic and with limited or without nociplastic components. Physical exam demonstrates functional lumbar instability, which conceptually differs from other structural etiologies of CLBP (discovertebral pain, facetogenic pain, stenotic pain). Ultrasonography and EMG may be helpful to assess multifidus activity with functional movements, while MRI is used to grade multifidus atrophy and assess for other structural pathologies that may be present concurrently with multifidus dysfunction and could be amenable to surgery and not to restorative neurostimulation.

Restorative neurostimulation is the only FDA-approved PNS device to treat CLBP associated with multifidus dysfunction and loss of neuromuscular control resulting in functional lumbar instability. Importantly, restorative neurostimulation distinguishes itself from traditional neurostimulation in a way that treats a different etiology, targets a different anatomical site, and has a distinctive MOA. Restorative neurostimulation is a novel disease-modifying therapy that through a rehabilitative MOA with efferent neurostimulation of the dorsal rami of the LMNB elicits repetitive multifidus contraction that with a gradual and longitudinal accrual time to effect has been proposed to override underlying AMI and restore mechanonociceptive feedback, which may normalize neuromuscular control and functional spinal stability. This therapy has been shown to reduce pain and disability in CLBP, improve quality of life and reduce health care expenditures.
